# Editorial: Assessing and addressing health inequities and disparities: The role of health informatics

**DOI:** 10.3389/fpubh.2023.1161892

**Published:** 2023-03-21

**Authors:** Gulzar H. Shah, Anjum Khurshid, Joanne Chopak-Foss

**Affiliations:** ^1^Jiann-Ping Hsu College of Public Health, Georgia Southern University, Statesboro, GA, United States; ^2^Department of Population Medicine, Harvard Medical School and the Harvard Pilgrim Health Care Institute, Boston, MA, United States

**Keywords:** health inequities, health disparities, health informatics, media use, mental health, older adults

Eliminating health equity and resulting health disparities are at the core of modern population health approaches. Health informatics has been increasingly acknowledged as an essential tool in achieving the desired population health outcomes, but the protective role of informatics may become questionable in the absence of sound scientific evidence about the design and implementation of informatics policies and approaches shown to practically advance the population health and patient-centered healthcare for marginalized population groups (1). The collection of articles in this special topic issue titled *Assessing and Addressing Health Inequities and Disparities: The Role of Health Informatics*, comprised an assortment of topics focusing on the role of various components and processes of health informatics relevant to disparities and inequities in health, directly or as intervening factors. The intent is to showcase health informatics research and empirical evidence that will be instrumental in promoting equitable population health and healthcare outcomes.

The importance of addressing health inequities is increasingly recognized in modern strategies for improving healthcare and public health outcomes. At the same time, improving health and eliminating health disparities requires equitable access to socioeconomic resources known as social determinants of health (SDoH) including assets and income, affordable education, healthy food, clean water, and means to pursue healthy lifestyles. The SDoH data are increasingly used to guide health equity assurance efforts in public health. Recent interest in population health by the healthcare industry and advances in healthcare informatics have fueled their ability to incorporate real-world SDoH data into healthcare information systems to enable EHR-user education and targeted workflow integration (2).

This special topic collection of research studies includes one systematic review and 10 empirical research studies based on primary or secondary data sources. The collection showcases an assortment of geographic coverage, the functionality of informatics, and outcomes. Studies use multi-country data (Qu et al.) as well as national to sub-national geographic coverage. Several studies covered the use of information systems, information technology, and data science in addressing health inequities and disparities in accessing healthcare services by individuals or efficiencies in implementing clinical solutions. The topics and research implications ranged from addressing inequities in access to preventative dental service (Qu et al.), the use of social media during this pandemic (Tegegne et al.), the impact on care coordination by professionals and patients' information exchange (Tegegne et al.; Zhang and Zhang), use of telehealth services for improvements in healthcare services in underserved areas, and in turn, reducing inequities in the distribution of medical resources (Gao et al.; Ganjali et al.), the impact of media use on disparities in physical and mental health among the older people (Wang et al.), and use of modern electronic health records for collection of data for measuring health equity and outcomes for population health (Pesel et al.). Some other studies highlighted the shortcomings of health informatics, including the negative impact of exclusively relying on social media for health communication while excluding traditional media such as TV (Pesel et al.) and the limitation of traditional surveillance systems that do not include data on social determinants of health (Yu et al.). One of the studies tackled best practices in health information system design, with the implications for efficiency in medical resources, improvements in disparities in access to care, improvements in doctor-patient communications, and efficient care coordination (Li et al.). Another study highlighted the lack of policy support in health information systems in health services (Herawati et al.). One of the studies did not establish the linkage between informatics and health equity, though indirect implications can be inferred. The study examined the role of artificial intelligence and machine learning in the individualized prediction of risk factors for high blood loss in the perioperative period of thoracolumbar burst fracture (TBF), which in turn improves clinicians' decision-making and perioperative management (Yang et al.).

In investigating the global effect of COVID-19 mitigation strategies on access to preventable dental care, Qu et al. demonstrated that the COVID-19 mitigation measures in countries across the world created conditions that resulted in an overall decline in the oral health status of people. More critically though, inequities in access the preventive dental care resources worsened the existing disparities in the utilization of dental care. The study findings implied that while containing a pandemic such as COVID-19 can require imposing restrictions that limit people's access to dental care, equitable access to socioeconomic resources becomes even more critical to avoid the spill-over effect of the pandemic on oral health disparities (Qu et al.). Zhang and Zhang used the theory of planned behavior to examine the factors affecting patients' opt-in intention for health information exchange. Detecting health inequities in access to care upstream through data exchanged by health information exchanges (HIEs) can be instrumental in preventing health outcome disparities.

Social media is increasingly considered a valuable and integral part of health informatics, particularly in a pandemic when the timely exchange of accurate information is extremely valuable in curbing the spread of viruses such as SARS-CoV-2. Tegegne et al. studied health professionals' attitudes toward the use of social media for COVID-19-related information. With their study setting in Bahir Dar City public health centers in Ethiopia, they assessed the health professionals' use of social media for COVID-19-related information, concluding that the level of use was moderate. The need for training, education, behavioral change, and knowledge about the usefulness of social media among professionals was emphasized. In addition, enabling factors such as developing trusted social media pages, and social media platforms were recognized to promote the use of social media for COVID-19-related information among health professionals.

Studies have examined the potential of telemedicine in acting as an equalizer in access to care for traditionally underserved populations, and is increasingly gaining popularity in the COVID-19 era. Gao et al. examined telemedicine in China, examining national and regional level necessity, development history, scale and coverage, and operational procedure involving telemedicine. Ganjali et al. conducted a systematic review to study the use of telehealth services for specific functions during the pandemic. They find that telehealth services positively impact patient outcomes in both emergency and outpatient settings. Counseling was the most common functionality observed in the selected studies, followed by monitoring and diagnosing. The authors conclude that telehealth could be effectively adopted in a health emergency, but further research is needed to identify characteristics of successful systems and relevant health outcomes for measurement. The theme emerging from the papers points to the importance of building health informatics systems in public health and population health to capture, share, measure, and analyze data related to inequity as a starting point to address health inequities. Our current systems do a poor job of being able to do so consistently and hence the importance of the examples highlighted in this issue. The examples range from measuring oral healthcare needs in 17 countries, developing electronic patient record systems in Italy, social media impact in Ethiopia and China, and assessing telehealth services during a pandemic. These papers also highlight the challenges of studying socioeconomic, behavioral, and environmental factors, collectively called SDoH, that impact health inequities but the data linking these factors to specific health outcomes in populations is not easily available or collected. This issue is therefore also a call to action to improve the ability to collect, analyze and use richer data using health informatics tools and research to develop better strategies to narrow health equity gaps in the population (Ganjali et al.).

Wang et al. use quantitative methods with a national data sample from the 2017 China General Social Survey. They study the role of media use on the physical and mental health of older adults while controlling for other factors. They find that health disparities due to educational levels which are seen in older adults are narrowed by the use of traditional media but contrarily increase due to internet use. Wang et al. recommend repeated health campaigns through traditional media, like TV, because of its higher use. They also suggest integrating public health messages with media campaigns and advocating policies for narrowing the digital gap to narrow the information gap among older adults.

Pesel et al. present an evaluation of a new electronic patient record system in the Italian healthcare system. The study reminds us of the fact that the digitization of patient records is not a universal phenomenon but one that is a fundamental step in measuring health equity and outcomes for population health (Pesel et al.). In their population-based study of older adults with lung cancer, Yu et al. use cancer surveillance data to develop predictive models for older adults undergoing early-lung cancer treatment. The study is a good example of the use of data for specific subpopulations, like older adults, but also highlights the limitations of surveillance data in not including socioeconomic factors that are important to understand lung cancer prognosis (Yu et al.).

Herawati et al. describe the problems experienced by the health information system in health services in Indonesia in getting support for policy-making. To effectively address the problems, the study proposed to model a mathematical concept for implementing the health information system in the implementation of JKN in Indonesia. A structural equation model with Lisrel 88 software was used to model the health information system. Structured input components such as governance, human resources, infrastructure, types of information system (IS)(program, JKN, management), and financing; process components: funding, technical guidance, and verification and validation; and output components: open access, standards and quality, utilization, bridging, and security. The concept for strengthening the health information system prioritizes improving the output components (standards, utilization, bridging, open access, and security) in the process components (funding, verification, technical guidance) while the input components (financing, human resources, governance, IS programs, infrastructure, IS JKN, IS management) were included in the structural equation model.

Overall, the collection of articles for this special topic was fairly aligned with the call for papers announcement. However, to our surprise, studies in this collection were predominantly concerned with equitable access to healthcare, quality of healthcare, and improvements in healthcare interventions, and the role of information technology and information systems in addressing promoting these aspects of healthcare. There remained a dearth of studies on the role of health informatics in addressing inequities in public health and population health outcomes. A future call for a special topic may want to solicit studies to address this gap. Anyway, the research evidence generated by the collection of articles in this special topic issue concerning the ways health information technology (HIT) can be leveraged to support improvements in healthcare outcomes and health equity is particularly important in this era of emerging public health threats and patient-centric, evidence-based healthcare.

## Author contributions

All authors listed have made a substantial, direct, and intellectual contribution to the work and approved it for publication.
